# Enhancement of Salinity Tolerance during Rice Seed Germination by Presoaking with Hemoglobin

**DOI:** 10.3390/ijms12042488

**Published:** 2011-04-12

**Authors:** Sheng Xu, Bing Hu, Ziyi He, Fei Ma, Jianfei Feng, Wenbiao Shen, Jie Yang

**Affiliations:** 1 Laboratory Centre of Life Sciences, College of Life Sciences, Nanjing Agricultural University, Nanjing 210095, China; E-Mails: 2008216034@njau.edu.cn (S.X.); njhubing@sina.com (B.H.); njheziyi@sina.com (Z.H.); 2010116135@njau.edu.cn (F.M.); 1328207@njau.edu.cn (J.F.); 2 Institute of Food Crops, Jiangsu Academy of Agricultural Sciences, Nanjing 210014, China

**Keywords:** heme oxygenase-1, hemoglobin, salinity tolerance, seed germination, *Oryza sativa*

## Abstract

Salinity stress is an important environmental constraint limiting the productivity of many crops worldwide. In this report, experiments were conducted to investigate the effects of seed presoaking by bovine hemoglobin, an inducer of heme oxygenase-1 (HO-1), on salinity tolerance in rice (*Oryza sativa*) plants. The results showed that different concentrations of the hemoglobin (0.01, 0.05, 0.2, 1.0, and 5.0 g/L) differentially alleviated the inhibition of rice seed germination and thereafter seedling shoot growth caused by 100 mM NaCl stress, and the responses of 1.0 g/L hemoglobin was the most obvious. Further analyses showed that application of hemoglobin not only increased the *HO-1* gene expression, but also differentially induced catalase (CAT), ascorbate peroxidase (APX), and superoxide dismutase (SOD) activities or transcripts, thus decreasing the lipid peroxidation in germinating rice seeds subjected to salt stress. Compared with non-hemoglobin treatment, hemoglobin presoaking also increased the potassium (K) to sodium (Na) ratio both in the root and shoot parts after salinity stress. The effect is specific for HO-1 since the potent HO-1 inhibitor zinc protoporphyrin IX (ZnPPIX) blocked the positive actions of hemoglobin on seed germination and seedling shoot growth. Overall, these results suggested that hemoglobin performs an advantageous role in enhancement of salinity tolerance during rice seed germination.

## 1. Introduction

Seed germination is a complex process involving various physical and biochemical cues such as water, light and phytohormones. It has been widely reported that seed germination as well as seedling growth are sensitive to various abiotic stresses. A growing body of evidence also showed that the inhibitory effect of salt stress on seed germination is alleviated by phytohormones, including ethylene [1], cytokinin (CTK) [2]), gibberellin acid (GA) [3], and messenger molecules such as hydrogen peroxide (H_2_O_2_) [4], nitric oxide (NO) [5], and carbon monoxide (CO) [6]. Meanwhile, high salt concentrations could cause ion imbalance and hyperosmotic stress in plants. Additionally, secondary stresses often occur, such as oxidative stress due to the overproduction of reactive oxygen species (ROS) in salt stressed plants [7]. To quench ROS, plant cells possess both enzymatic and non-enzymatic mechanisms for the scavenging of ROS overproduction. It has been well established that the coordination activities of the multiple forms of antioxidant enzymes, such as superoxide dismutase (SOD), catalase (CAT), ascorbate peroxidase (APX) in the different cell compartments, achieve a balance between the rate of formation and the scavenging of ROS, and maintain ROS at suitable levels required for cell signaling.

Heme catabolism in a wide range of species is mediated by the heme oxygenase family of enzymes (HOs; EC 1.14.99.3). In animals, HOs are located within the endoplasmic reticulum, where they serve, in concert with NADPH cytochrome P450 reductase, to oxidize heme to biliverdin, free ferrous iron, and CO. It is well known that HOs exist in three major isoforms: HO-1, an inducible form, and HO-2/3 belonging to constitutively expressed isozymes. Further results in plants have confirmed that a plastidic HO-1 in Arabidopsis was the potential source of CO as well [8], and its role in the participation in the biosynthetic pathway leading to phytochrome chromophore formation and/or light signaling were also reported [9]. Recent reports confirmed that HO could act as a strong antioxidant enzyme against various abiotic stresses, as well as the inducer functioning in different developmental processes, including adventitious and lateral root development [10,11].

In animals, oxidative stress-increased HO-1 protein has been well established. Further ample observations demonstrated that increased endogenous HO-1 provided cellular protection against oxidant injury [12]. Hemoglobin and its catalyzed products including hemin were able to induce the expression of HO-1 in cerebral arterial smooth muscle during the 24-h period in a time- and dose-dependent mode [13], suggesting that the induction of HO-1 in mammalian cells is a general response to heme-containing compounds and probably to oxidant stress which results from the oxidation of ferrous to ferric hemoglobin [14]. Hemoglobin was able to induce the lung HO-1 expression, which plays an important role in the defense against I/R-induced lung injury [15]. In plants, it was demonstrated that the supplementation of culture medium with bovine hemoglobin clearly promoted mitotic division of protoplasts in a *japonica* rice variety, Taipei 309. Meanwhile, an increase in shoot regeneration from protoplast-derived tissues also occurred [16]. However, whether hemoglobin could be used in seed pretreatment to induce salt tolerance is still unknown.

It is well known that better understanding of mechanisms(s) that enable plants to adapt salt stress is necessary to make the best use of saline soils. In this report, we investigated some physiological and biochemical events induced by bovine hemoglobin presoaking, including the alleviation of seed germination and growth inhibition, lipid peroxidation, re-establishment of ironic unbalance, and the up-regulation of antioxidant enzyme expression. The possible mechanisms of hemoglobin involved in conferring salt tolerance were preliminarily discussed.

## 2. Results

### 2.1. The Inhibition of Seed Germination and Seedling Shoot Growth Were Alleviated by the Pretreatment of Hemoglobin

In comparison with the control treatment (H_2_O→H_2_O), rice seed germination and seedling growth were inhibited significantly by 100 mM NaCl salt stress treatment (H_2_O→S, Table 1). Further results showed that the different concentrations of hemoglobin pretreatment were able to reverse the negative impact of NaCl on seed germination and shoot growth inhibition, with a maximal response at 1.0 g/L hemoglobin (Hb1.0→S). For example, when compared to the NaCl-treatment alone sample, the addition of 1.0 g/L hemoglobin resulted in the increment of 48.0% and 32.9% in seed germination and shoot length, respectively. Meanwhile, the slight negative effects on root length appeared when the higher doses of hemoglobin (0.2, 1.0, and 5.0 g/L) were adopted. Further parallel experiments suggested that, except the aggravation of root growth inhibition conferred by 5.0 g/L hemoglobin, no significant differences in seed germination and seedling growth were observed in the hemoglobin pretreatment alone, compared to salt stressed sample.

To test the hypothesis that HO-1 was involved in above hemoglobin-induced responses, the potent HO-1 inhibitor ZnPPIX proven in both animals and plants [17,18] was applied. In our experiment, the application of ZnPPIX prevented the alleviation actions of hemoglobin on the inhibitions of seed germination (especially) and shoots growth caused by salt stress (Table 2). In contrast, only a slight but not significant decrease could be observed in response to the addition of ZnPPIX together with NaCl (H_2_O→S + ZnPPIX) compared with NaCl stressed alone sample (H_2_O→S). The above results strongly suggested the role of HO-1 in hemoglobin-induced cytoprotective roles.

### 2.2. Lipid Peroxidation and ROS-Scavenging Enzyme Activities

**Figure 1.**
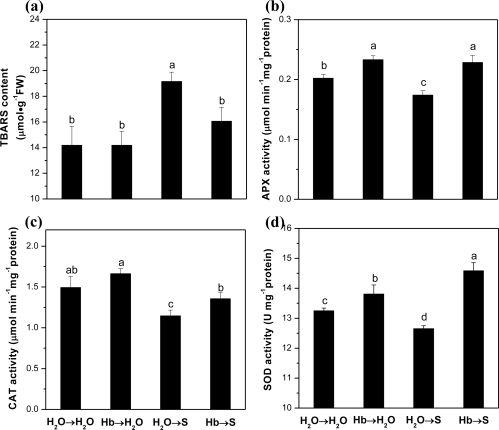
Effects of hemoglobin pretreatment on the TBARS content **(a)**, APX **(b)**, CAT **(c)**, and SOD **(d)** activities in rice germinating seeds upon salt stress. Dry rice seeds were presoaked in distilled water or 1.0 g/L Hb for 1 d, and then shifted to 100 mM NaCl solution for another 60 h. Sample with distilled water treatment alone (H_2_O) was used as the control. Data are the means ± SE of at least three independent experiments. Bars denoted by different letters were significantly different at *P* < 0.05 according to a Duncan’s multiple comparison.

In a subsequent experiment, TBARS contents of rice germinating seeds subjected to 100 mM NaCl with or without hemoglobin pretreatment were compared. It was found that in comparison with the control, TBARS content was enhanced by about 35.0% upon NaCl stress alone, but less than 13.1% in rice seeds exposed to hemoglobin pretreatment followed by the addition of NaCl (Figure 1a). Additionally, no significant difference in TBARS was observed between hemoglobin applied alone (Hb→H_2_O) and control samples (H_2_O→H_2_O).

It was well known that APX, CAT, and SOD enzymes detoxify ROS in plant tissues. Thus, their activities were determined as representative enzymes responsible for antioxidant metabolism. The results shown in Figure 1b–d showed that with respect to the control samples, the activities of all above three enzymes decreased significantly upon salinity stress. While, the response patterns of the samples subjected to hemoglobin pretreatment were opposite. For example, APX, CAT, and SOD activities increased after 60 h of salinity stress, being 31.3%, 18.3%, and 15.3%, respectively, higher than that in the sample under salinity stress alone. Similarly, the inducible responses were also observed when hemoglobin was preincubated alone in comparison with the control sample.

### 2.3. Antioxidant Enzyme Transcripts

**Figure 2.**
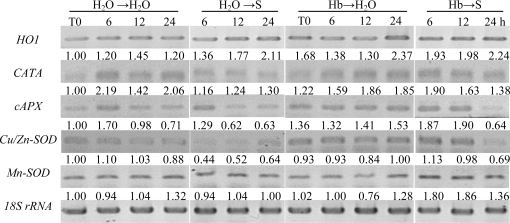
Effects of hemoglobin pretreatment on the transcription levels of *HO1*, *CATA*, *cAPX*, *Cu/Zn-SOD*, and *Mn-SOD,* in rice germinating seeds upon salt stress. Dry rice seeds were presoaked in distilled water or 1.0 g/L Hb for 1 d, and then shifted to 100 mM NaCl solution for another 0, 6, 12, and 24 h. Sample with distilled water treatment alone (H_2_O) was the control. The numbers below the band indicate relative abundance of corresponding transcripts with respect to the data at time zero (T0).

Semi-quantitative RT-PCR was applied to investigate the responses of antioxidant enzyme transcripts upon different treatments. Time-course experiment showed that the pretreatment of hemoglobin could significantly strengthen the inducible effect of salinity on the up-regulation of HO-1 transcripts through 24 h of incubation (Figure 2). Meanwhile, in comparison with the NaCl-free control, the amount of the *CATA*, *cAPX* and *Cu/Zn-SOD* transcripts in NaCl-treated sample was decreased. While, no significant changes were observed in the transcripts of *Mn-SOD*. Furthermore, pretreatment with hemoglobin led to significant increases in above transcripts compared with samples without corresponding pretreatment before NaCl treatment. We also noticed that above changes in mRNA levels approximately coincided with the changes of the corresponding CAT, APX, and SOD activities (Figure 1b–d).

### 2.4. Changes of K/Na Ratio

**Figure 3.**
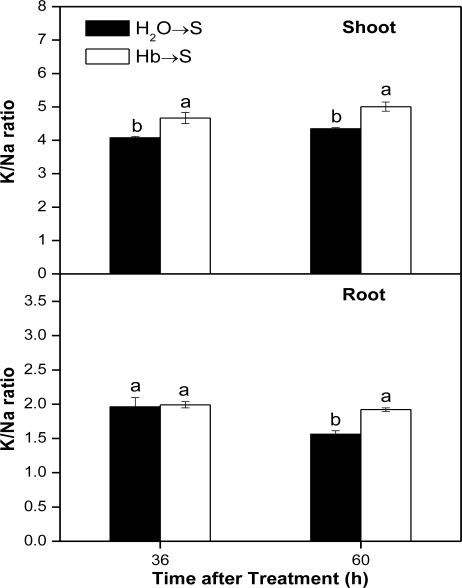
Effects of hemoglobin pretreatment on K/Na ratio in shoot and root parts from rice seedlings under salt stress. Dry rice seeds were presoaked in distilled water or 1.0 g/L Hb for 1 d, and then shifted to 100 mM NaCl solution for another 36 h and 60 h; Data are the means ±SE of at least three independent experiments. Bars denoted by different letters were significantly different at *P* < 0.05 according to the Duncan’s multiple comparison.

**Figure 4.**
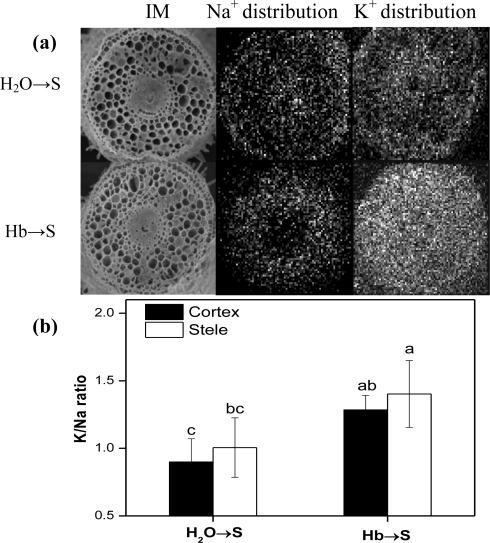
Effects of hemoglobin pretreatment on K and Na element distribution in root parts of rice seedlings under salt stress. Dry rice seeds were presoaked in distilled water or 1.0 g/L Hb for 1 d, and then shifted to 100 mM NaCl solution. X-ray micrography of root tips of rice seedlings treated for 60 h, the enrichment of K or Na distribution shown with white dots **(a)**; IM, scanning electron microscope (SEM) image of corresponding section; Scale bar = 100 μm; K/Na ratio detected in the cortex and stele of root tips **(b)**. Data are the means ± SE of at least three independent experiments. Bars denoted by different letters were significantly different at *P* < 0.05 according to the Duncan’s multiple comparison.

The ionic balance inside the plant cell is closely related to the plant adaptation to salt stress, so we compared K/Na ratio in both the whole shoot and root parts and especially the root tips from seedlings under salt stress. Figure 3 showed that the inducible changes of K/Na ratio were observed in shoot (particularly) and root tissues of hemoglobin pretreatment samples after NaCl treatment for 36 h and 60 h, suggesting that hemoglobin pretreatment could enhance salinity tolerance by re-establishing the ion homeostasis. Furthermore, the X-ray density maps of the distribution of Na and K elements in root tips of rice seedlings at 60 h after salinity treatment showed that in comparison with these of salt-stressed alone samples, more K but less Na were observed in the root tip sections of sample pretreated with hemoglobin (Figure 4a), thus resulting in the enhancement of the K/Na ratio, especially in cortical cells of root tips (Figure 4b).

## 3. Discussion

Exogenous use of various chemicals to reduce the adverse effects of abiotic stresses has great implications both from theoretical and practical perspectives [19]. In our test, soaking of rice seeds in increased hemoglobin levels (from 0.01 to 5.0 g/L) for 24 h could differentially modulate seed germination and seedling shoot growth under salt stress in rice plants (Table 1), and the responses of 1.0 g/L hemoglobin was the most obvious. To the best of our knowledge, above observations are new. In fact, similar inducible responses conferred by bovine hemoglobin have been observed in the mitotic division and plant regeneration from cultured rice protoplasts [16], and the growth of cultured cotton cells [20].

Use of hemoglobin in seed presoaking is advantageous only when it supports the emergence and establishment of seedlings. Initial absorption and later scavenging of ROS in the rice seeds provided evidence about the occurrence of changes mainly related to the protection against oxidative damage. Therefore, we investigated the effect of hemoglobin produced changes on the lipid peroxidation and the expression of antioxidant enzymes in salinized hemoglobin-pretreated seedlings of rice and compared with water control and salinized alone samples. Further results showed that 1.0 g/L hemoglobin was able to counteract the increases in TBARS content induced by salinity in germinating rice seeds (Figure 1a), and this effect may be attributed to the ability of hemoglobin to activate antioxidant enzymes, including SOD, CAT, and APX (Figure 1b–d). Transcription levels of corresponding genes were also up-regulated (Figure 2). These findings were consistent with those reported by Garratt *et al.* [20], in which researchers found SOD activity increased linearly with the increasing concentrations of Erythrogen, a commercial bovine hemoglobin, in cultured cotton cells. Meanwhile, higher concentration of Erythrogen could bring about a concomitant increase in CAT activity to a maximum of 62% more than the control sample.

Another important mechanism regarding salt stress in plants is attributed to ionic phyto-toxicity caused by excess amounts of salt ions in plants. Thus, re-establishment of ion homeostasis is essential for plants to resist salt stress. Here, the application of hemoglobin presoaking treatment was able to induce the increment of K/Na ratio in salt stressed seedling shoots and roots or even root tips (Figure 3 and Figure 4). Thus, ion homeostasis is re-established so as to adapt the plant cell to salt stress.

Expression of stress protein is an important adaptive strategy of environmental stress tolerance. Normally, they are highly water soluble and heat stable. Our previous report showed that salinity stress could induce HO activity in wheat seedling roots, and the product of HO, CO was able to enhance salt tolerance by NO-mediated maintenance of ion homeostasis and up-regulation of antioxidant defense [21]. In this test, we also noticed that 1.0 g/L hemoglobin pretreatment followed by salinity stress can effectively strengthen the up-regulation of HO-1 gene expression induced by salinity stress alone treatment (Figure 2a). Similar inducible response of HO-1 driven by hemoglobin was discovered in animals previously [13–15]. Combined with the fact that the addition of ZnPPIX notably prevented the inducible action of hemoglobin on rice seed germination and seedling shoot growth (Table 2), it is speculated that the up-regulation of HO-1 expression might contribute to the responses of hemoglobin. Previously, we discovered that CO, one of the product of HO-1, played an important role against salinity- or osmotic-induced oxidative cell damage by enhancing antioxidant system parameters in wheat [22,23] and rice seedlings [6]. In wheat alurone cells, the up-regulation of HO-1 expression delayed programmed cell death (PCD) by the down-regulation of H_2_O_2_ production [24]. In view of the fact that hemoglobin released from senescing erythrocytes has been confirmed as the resource of CO synthesis as early as in 1949 [25], it is interesting and necessary in the future to investigate the specific role of HO-1/CO in the above hemoglobin-induced plant physiological processes.

## 4. Experimental Section

### 4.1. Chemicals

Commercial bovine hemoglobin, purchased from Shanghai Boao Ltd., China, was used as an HO-1 inducer. Zinc protoporphyrin IX (ZnPPIX), a specific inhibitor of HO-1 [17,18], was obtained from Sigma (St Louis, MO, USA) and used at 100 μM.

### 4.2. Plant Material and Experimental Design

For the presoaking experiments, rice (*Oryza sativa* L., Wuyujing 7) seeds, kindly supplied by Jiangsu Academy of Agricultural Sciences, Jiangsu Province, China, were presoaked in 0.01, 0.05, 0.2, 1.0, and 5.0 g/L commercial bovine hemoglobin or distilled water as a control (Con). All seeds were incubated in a growth chamber (Shanghai Yiheng Technology Co., Ltd., Shanghai, China) at 30 °C under darkness condition for 24 h. Then, seeds were placed in petri dishes containing filter paper wet with 6 mL of distilled water (Con), or 100 mM NaCl, 100 μM ZnPPIX, or its combination treatment, and also incubated in a similar growth chamber. After 60 h or at the indicated time point of salinity treatment, the samples were harvested, growth parameters were determined, and corresponding materials were frozen at −80 °C for further analysis.

### 4.3. Germination and Growth Analysis

Germination tests were performed on three replicates of 150 seeds each. There were 50 seeds in each petri dish. Cumulative germination rate (%) was recorded at 60 h after salinity treatment, and seeds were considered to have germinated when the emerging shoot was approximately half the length of the seeds. Meanwhile, the measurements of root length and shoot length were carried out after different treatments.

### 4.4. TBARS Determination

Oxidative damage was estimated by measuring the concentration of TBARS as described previously [26].

### 4.5. Antioxidant Enzyme Assays

Frozen rice plants (approximately 200 mg) were homogenized in 10 mL of 50 mM potassium phosphate buffer (pH 7.0) containing 1 mM EDTA and 1% polyvinylpyrrolidone (PVP) for CAT, and SOD assay, or combination with the addition of 1 mM ascorbic acid (ASC) in the case of APX assay. The homogenate was centrifuged at 12,000 g (Avanti J-25, Beckman) for 20 min at 4 °C and the supernatant was desalted immediately by Sephadex G-25 gel filtration to remove interfering materials and used as the crude enzyme extract.

APX activity was measured by monitoring the decrease in absorbance at 290 nm as ASC was oxidized (ɛ = 2.8 mM^−1^ cm^−1^) for at least 1 min in 3 mL reaction mixture, as described by Nakano *et al.* [27]. CAT activity was spectrophotometrically measured by monitoring the consumption of H_2_O_2_ (ɛ = 39.4 mM^−1^ cm^−1^) at 240 nm for at least 3 min [22]. Total superoxide dismutase (SOD) activity was measured on the basis of its ability to reduce nitroblue tetrazolium (NBT) by superoxide anion generated by the riboflavin system under illumination. One unit of SOD (U) was defined as the amount of crude enzyme extract required to inhibit the reduction rate of NBT by 50% [28]. Protein concentration was determined by the method of [29], using bovine serum albumin (BSA) as a standard.

### 4.6. Semi-Quantitative RT-PCR Analysis

100 mg fresh rice tissue was homogenized by grinding with mortar and pestle in liquid nitrogen, and total RNA was isolated using Trizol reagent (Invitrogen) according to the manufacturer’s instructions. DNA-free total RNA (5 μg) from different samples were used for first-strand cDNA synthesis in a 20-μL reaction volume containing 2.5 units of avian myeloblastosis virus reverse transcriptase XL (TaKaRa) and 2.5 μM random primer. PCR was performed using 2 μL of a 20-fold dilution of the cDNA, 10 pmol of each oligonucleotide primer, and 1 unit of Taq polymerase (TaKaRa) in a 25-μL reaction volume.

cDNA was amplified by PCR using the following primers: for rice *HO-1* (accession number EU781632), forward (5′-AGGAATACTGGGTTGGAGAG-3′) and reverse (5′-GCTTAGGTGAATATGTGACGGA-3′), amplifying a 416-bp fragment; for *CATA* (accession number AK061923), forward (5′-AGGCCAGACAATGTCAGATG-3′) and reverse (5′-GTGGCATTAATACGCCAGTA-3′), amplifying a 202-bp fragment; for *cAPX* (accession number AK061841), forward (5′-CATTGCCCGTGGTACTCT-3′) and reverse (5′-TTTCATACCAACACATCT-3′), amplifying a 199-bp fragment; for *Cu/Zn-SOD* (accession number L36320), forward (5′-AATGGTGAAGGCTGTTGTTGT-3′) and reverse (5′-TAGCCTTGAAGTCCGATGA-3′), amplifying a 461-bp fragment; for *Mn-SOD* (accession number L34038), forward (5′-CCAGAAGCACCACGCCACCTAC-3′) and reverse (5′-CTCCCAGACATCAATTCCCAAC-3′), amplifying a 418-bp fragment; and for *18S rRNA* (accession number AK059783), forward (5′-TACCGTCCTAGTCTCAACCA-3′) and reverse (5′-AGAACATCTAAGGGCATCACA-3′), amplifying a 451-bp fragment. To standardize the results, the relative abundance of *18S rRNA* was determined and used as the internal reference.

The cycle numbers of the PCR reactions were adjusted for each gene to obtain clear bands in agarose gels. Aliquots of the PCR reactions were loaded in 1.2% agarose gels with ethidium bromide. Specific amplification products of the expected size were observed, and their identities were confirmed by sequencing.

### 4.7. Determination of Ion Contents

Na and K contents of shoot and root parts were measured by an atomic emission spectrophotometer (TAS-986; Beijing Purkinje General Instrument Co., Ltd., Beijing, China).

### 4.8. X-ray Microanalysis

Element ratio measurement was examined with a scanning electron microscope (SEM) (Model S-3000N, Hitachi High-Technologies Corporation, Japan) equipped with an energy-dispersive X-ray detector (EDX, Horiba Inc. Kyoto, Japan) as described previously [30,31] with some modifications. After being placed vertically in the hole of an aluminum stub and immersed quickly into liquid nitrogen for freeze-drying, the tender root tips were transferred quickly into a vacuum evaporator and dried under the vacuum of 5 × 10^−4^ Torri for at least 12 h. The root tips were coated with a fine layer of pure evaporated carbon and then observed. Acceleration voltage of 10 kV was used. Beam current was adjusted to a fixed (0.06 μA), and the working distance from the EDX detector was 13.5 mm. For analyses of relative elemental levels within cortical and stelar cells of root tips accurately, point analysis of each sample was repeated at least four times. The results were calculated by expressing the atomic number for a particular element in a given point or region as a percentage of the total atomic number for all the elements measured (K, Na, calcium, magnesium, phosphorus, sulfur, and chlorine) in the root tips.

### 4.9. Statistical Analysis

All results are expressed as mean values ± SE of at least three independent experiments. For statistical analysis, Duncan’s multiple test was used as appropriate, after testing for data normality. A value of *P* < 0.05 was considered significant for mean differences.

## 5. Conclusions

Hemoglobin attenuated salinity-induced inhibition of rice seed germination and seedling shoot growth. It was partially due to the induction of antioxidant metabolisms and reestablishment of ion homeostasis. Such responses may be of considerable value in the development of improved methods for crop protection against environmental stresses. Interestingly, our results in rice seeds correspondingly demonstrated that the effects of hemoglobin functions in animal research, such as achieving cytoprotective function, also work in plants.

## Figures and Tables

**Table 1.** Effects of different concentrations of hemoglobin (Hb, 0.01–5.0 g/L) pretreatments for 24 h on the inhibition of rice seed germination, and seedling shoot and root length caused by 100 mM NaCl stress for another 60 h.

**Treatment**	**Germination rate (%)**	**Shoot length (cm)**	**Root length (cm)**
H_2_O→H_2_O	89.55 ± 2.52^a^	1.34 ± 0.10^a^	3.44 ± 0.05^a^
Hb0.01→H_2_O	88.32 ± 2.84^a^	1.37 ± 0.11^a^	3.39 ± 0.25^a^
Hb0.05→H_2_O	90.51 ± 2.05^a^	1.35 ± 0.08^a^	3.35 ± 0.07^a^
Hb0.2→H_2_O	95.44 ± 0.69^a^	1.38 ± 0.11^a^	3.34 ± 0.04^a^
Hb1.0→H_2_O	96.34 ± 2.69^a^	1.44 ± 0.10^a^	3.27 ± 0.22^a^
Hb5.0→H_2_O	94.43 ± 2.11^a^	1.45 ± 0.05^a^	3.05 ± 0.20^b^
H_2_O→S	45.47 ± 7.26^c^	0.70 ± 0.05^c^	2.55 ± 0.25^bc^
Hb0.01→S	51.48 ± 3.44^c^	0.76 ± 0.10^bc^	2.80 ± 0.48^bc^
Hb0.05→S	52.86 ± 2.69^c^	0.76 ± 0.10^bc^	2.57 ± 0.51^bc^
Hb0.2→S	61.05 ± 9.67^b^	0.87 ± 0.10^b^	2.50 ± 0.29^c^
Hb1.0→S	67.31 ± 3.92^b^	0.93 ± 0.09^b^	2.47 ± 0.01^c^
Hb5.0→S	62.98 ± 5.47^b^	0.90 ± 0.03^b^	2.38 ± 0.24^c^

Values are the means ± SE of at least three independent experiments. Different letters in the same column indicate significant differences (*P* < 0.05) according to Duncan’s multiple test.

**Table 2.** Effects of hemoglobin (Hb, 1.0 g/L) pretreatment for 24 h on the inhibition of rice seed germination, and seedling shoot and root length, caused by 100 mM NaCl stress in the presence and absence of the HO-1 inhibitor ZnPPIX (100 μM) for another 60 h.

**Treatment**	**Germination rate (%)**	**Shoot length (cm)**	**Root length (cm)**
H_2_O→H_2_O	90.30 ± 2.38^a^	1.33 ± 0.20^a^	3.46 ± 0.14^a^
Hb1.0→H_2_O	94.47 ± 2.49^a^	1.46 ± 0.16^a^	3.30 ± 0.23^a^
H_2_O→ZnPPIX	87.55 ± 2.08^ab^	1.15 ± 0.24^ab^	2.90 ± 0.10^ab^
Hb1.0→ZnPPIX	84.54 ± 5.05^ab^	1.18 ± 0.25^ab^	3.04 ± 0.30^ab^
H_2_O→S	46.32 ± 6.59^d^	0.70 ± 0.08^c^	2.58 ± 0.33^b^
Hb1.0→S	64.88 ± 3.77^c^	0.90 ± 0.11^b^	2.47 ± 0.10^b^
H_2_O→S + ZnPPIX	44.19 ± 5.04^d^	0.66 ± 0.07^c^	2.14 ± 0.25^bc^
Hb1.0→S + ZnPPIX	45.70 ± 2.58^d^	0.79 ± 0.09^bc^	1.95 ± 0.21^c^

Values are the means ± SE of at least three independent experiments. Different letters within columns indicate significant differences (*P* < 0.05) according to Duncan’s multiple test.
